# Vertical Transmission of Hepatitis C Virus Among Women With a History of Injection Opioid Use

**DOI:** 10.1093/cid/ciae177

**Published:** 2024-04-05

**Authors:** John M Cafardi, Hong T Lin, Lana Lange, Lacey Kelley, Kelly Lemon, Elizabeth A Odegard, Heidi L Meeds, Jason T Blackard, Judith Feinberg

**Affiliations:** Division of Infectious Diseases, Department of Medicine, The Christ Hospital and the Lindner Research Institute, Cincinnati, Ohio, USA; Division of Digestive Diseases, Department of Internal Medicine, University of Cincinnati College of Medicine, Cincinnati, Ohio, USA; Department of Gynecology and Obstetrics, The Christ Hospital, Cincinnati, Ohio, USA; Department of Behavioral Medicine and Psychiatry, West Virginia University School of Medicine, Morgantown, West Virginia, USA; Department of Gynecology and Obstetrics, West Virginia University School of Medicine, Morgantown, West Virginia, USA; Division of Digestive Diseases, Department of Internal Medicine, University of Cincinnati College of Medicine, Cincinnati, Ohio, USA; Division of Digestive Diseases, Department of Internal Medicine, University of Cincinnati College of Medicine, Cincinnati, Ohio, USA; Division of Digestive Diseases, Department of Internal Medicine, University of Cincinnati College of Medicine, Cincinnati, Ohio, USA; Departments of Behavioral Medicine and Psychiatry & Medicine/Infectious Diseases, West Virginia University School of Medicine, Morgantown, West Virginia, USA

**Keywords:** hepatitis C, pregnancy, vertical transmission, phylogenetic analysis

## Abstract

We evaluated vertical transmission and linkage to care in women with hepatitis C virus (HCV) and history of injection drug use employing co-localized testing and treatment. Transmission occurred in 1 of 23 infants, with mother-infant genetic distance of 1.26%. Rates for infant testing, maternal linkage, and cure were 77%, 52%, and 100%, respectively.

Over the past 2 decades, there has been a marked escalation in the incidence of hepatitis C virus (HCV) infection in central Appalachia, primarily due to the high prevalence of injection drug use (IDU), along with inadequate access to healthcare [1]. Although there was a 22% increase in HCV among women of childbearing age nationwide between 2012 and 2014, Ohio had an 89% increase between 2010 and 2015 [1]. These increases have been observed nearly exclusively in rural, non-Hispanic White persons. Furthermore, the proportion of children born to women with HCV infection nationally rose 68% during this time [2].

A meta-analysis of 17 studies estimated the rate of vertical transmission in HCV monoinfection at 5.8% (95% confidence interval [CI]: 4.2%–7.8%) [3]. Transmission correlated with the magnitude of maternal HCV RNA, with no transmission observed when the viral load was <3.5 log_10_ IU/mL and rising to 11% with viral loads >6 log_10_ IU/mL [4]. Human immunodeficiency virus (HIV) coinfection increases the HCV transmission rate to 10.8% [3]. Historically, it was thought that vertical transmission occurs at or near the time of delivery; however, recent data suggest that transmission may occur between 25 and 36 weeks of gestation [5]. In addition to vertical transmission, maternal HCV infection is associated with adverse obstetric and neonatal outcomes including preterm labor, preterm delivery, low birth weight/small for gestational age, and neonatal intensive care unit admission [4].

Testing of exposed infants for HCV RNA between 2 and 6 months is advised, with repeat nucleic acid testing to confirm the diagnosis at 3 years of age prior to initiation of therapy, although follow-up has been poor historically [6]. Linkage to care in this population has also been inadequate, with 17% linked without intervention; however, this could be increased to 52% with implementation of outreach strategies [7]. Given the increasing prevalence of HCV among women of childbearing capacity, neonatal and obstetric morbidity, and limited linkage to care, an improved understanding of vertical transmission, and linkage to testing and treatment are needed.

We present the results of an observational study of HCV vertical transmission with post-natal infant testing out to 48 weeks and linkage to care and curative treatment postpartum in women with a history of injection drug use in southern Ohio and West Virginia (NCT03570112).

## METHODS

Fifty-four pregnant women with chronic HCV infection were recruited from outpatient clinics in Cincinnati, Ohio, and Morgantown, West Virginia. Eligible participants were at least 18 years old with singleton pregnancy up to 36 weeks. Exclusions included hepatitis B coinfection, active drug use, decompensated cirrhosis, and severe medical comorbidities. Well-controlled HIV coinfection (HIV RNA < 20 copies/mL) and gestational diabetes were permitted.

After obtaining written informed consent, participants were educated about chronic HCV infection and were scheduled for seven study visits including enrollment, 36 weeks gestation, and 12, 24, 36, and 48 weeks postpartum. IRB approval and informed consent forms are available from the authors upon request. All participants were offered once daily sofosbuvir-velpatasvir 400–100 mg (Epclusa®—Gilead Sciences, Foster City, California, USA) for 84 days beginning 24 weeks postpartum with cessation of breastfeeding and a negative pregnancy test confirmed prior to treatment. Three blood samples were collected from each participant at 36 weeks of gestation as well as at 28 weeks postpartum (4 weeks after starting HCV treatment) and 48 weeks postpartum (12 weeks after completing HCV treatment). Infant blood samples were collected at 12, 24, and 48 weeks following delivery. All maternal and infant testing was performed simultaneously at co-localized appointments.

HCV RNA was extracted from serum. Viral RNA was amplified by real-time polymerase chain reaction (PCR) with Murphy's 5' untranslated region (UTR) primers (antisense 5′—CTC GCA AGC ACC CTA TCA GG—3′ and sense 5′-GAA AGC GTC TAG CCA TGG CGT TAG T—3′) and SYBR Green probe. Each sample was run separately in 3 wells, and positive, negative, and no-template controls were included. The presence and quantity of HCV RNA were then confirmed through melting curve analysis. In samples that were HCV RNA positive, a partial region of the nonstructural protein 5B (NS5B) gene was amplified as previously described and next-generation sequencing (NGS) was performed. NGS reads were mapped to the X76918 reference, cropped to the NS5B region in UGENE v45.0, and trimmed to areas of full coverage [8]. Sequences were aligned to a set of references in Clustal X to determine the HCV genotype and relatedness of mother-infant samples [9]. A phylogenetic tree was generated with neighbor-joining method and 1000 bootstrap replicates. Genetic distance between the infected mother-infant pair was then calculated in MEGA11 using the Kimura 2-parameter model [10].

## RESULTS

Of the 54 pregnant women enrolled, data were available for 49 mothers and 30 infants. Not all infants were available for testing (7 due to loss of custody and 12 due to maternal follow-up without the infant present). All mothers were offered treatment regardless of infant follow-up status. All mothers had past histories of opioid injection drug use; none reported or showed clinical evidence of current illicit drug use during the study. Per self-reported data, 52 were White, and 2 were Black, consistent with previously observed racial distribution. One participant was coinfected with well-controlled HIV. All maternal samples were confirmed to be HCV RNA positive by real-time PCR. Due to maternal nonadherence to study visits, infant placement in foster care, and coronavirus disease 2019 (COVID-19)-related restrictions, only 23/30 (77%) of the evaluable infants were tested, yielding 23 mother-infant pairs. The screening rate of all live-born infants, including those for whom data were not available, was 47% (23/49). Of the infants for whom data were available, 5 (22%) completed all 3 study visits, 7 (30%) completed 2 visits, and 11 (48%) completed 1 visit.

Of mothers with evaluable data, 28/54 (52%) were successfully linked to care and completed sofosbuvir/velpatasvir treatment. All participants who started treatment had undetectable HCV RNA at the end of treatment. Twelve participants returned for sustained virological response at 12 weeks post-treatment (SVR12) assessment, and all achieved SVR12 (22% of total participants). The remaining 26 (48% of participants) did not begin therapy or did not return for HCV RNA testing. Follow-up was not affected by use of opioid agonist therapy; however, inconsistent follow-up limits the generalizability of this observation.

One infant tested positive for HCV RNA at the 24-week visit, yielding a vertical transmission rate of 4% (1 of 23). The maternal viral load prior to delivery was 2.1 million IU/mL and did not have HIV coinfection.

**Figure 1.**
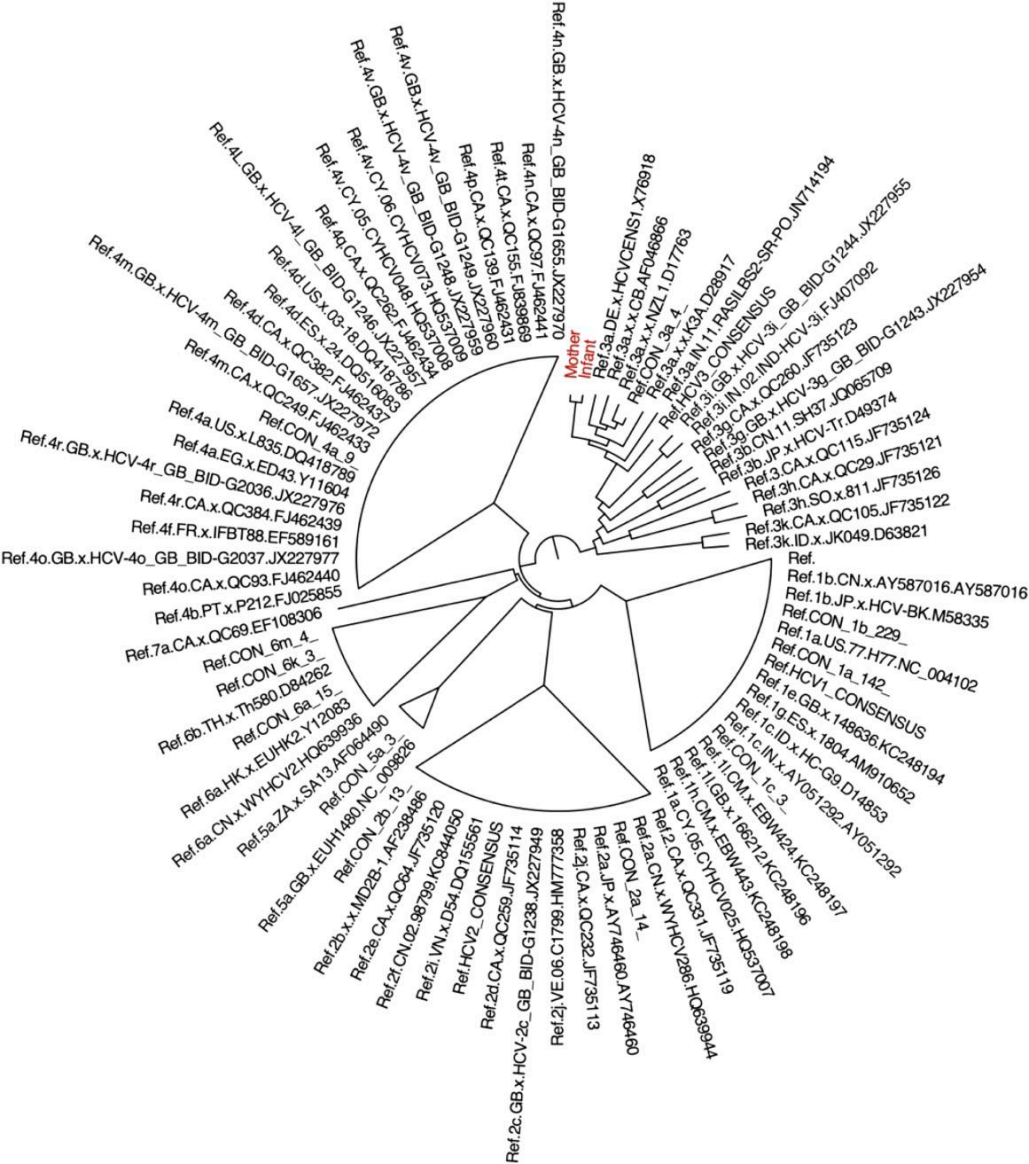
Phylogenetic tree generated from NGS of NS5B region fragments from the mother-infant HCV transmission pair. Abbreviations: HCV, hepatitis C virus; NGS, next generation sequencing; NS5B, nonstructural protein 5B.

Analysis of a ∼395 base pair fragment of the NS5B gene showed that the mother and infant were both infected with HCV genotype 3a (Figure 1). These 2 sequences were most closely related to each other compared to all other references. The genetic distance between the 2 samples was 1.26%, corresponding to a difference of 7 nucleotides. Although this region of NS5B includes several amino acid positions associated with viral fitness, replication and/or drug resistance, including R222, D225, Y276, S282, T287, N291, G317, D318, and D319, wild-type amino acids were observed in the infant and mother sequences at each of these positions.

## DISCUSSION

Although the transmission rate may be underestimated by incomplete testing and follow-up, the observed rate of 4% is consistent with previously described rates in HCV monoinfection [3]. Ongoing research into the treatment of HCV during pregnancy should provide important data about preventing vertical transmission.

Genetic diversity is a key feature of HCV. At the population level, HCV consists of multiple genotypes and subtypes, which may affect treatment response and influence disease pathogenesis. The HCV NS5B protein is an RNA-dependent RNA polymerase that lacks a proofreading mechanism. Sequencing of the NS5B is commonly performed to determine the HCV genotype and identify potential resistance-associated mutations [11].

Although factors such as HIV coinfection, HCV therapy, and immunologic pressure increase NS5B genetic distance within an individual [12], no studies currently exist describing NS5B variability in mother-infant pairs. Additional data may elucidate risk factors for increased genetic variability, transmissibility, and the potential development of drug resistance.

Co-localization of maternal and infant visits for testing as well as maternal treatment resulted in higher rates of linkage to care and maternal HCV treatment than previously described, although loss of custody and ensuing foster care placement, lack of transportation and relocation distant from the study sites, and the COVID-19 pandemic were significant impediments to both infant testing and maternal linkage to care. Generalization of these results would likely require significant investment in outreach, as research staff provided phone reminders and transportation assistance as needed to study participants before each appointment. Further development of programs offering co-localized testing and treatment of pregnant women with HCV and their infants is a promising strategy for linkage to care and treatment.

## Notes

***Financial support***. This work was supported by Gilead Sciences, Foster City, CA 94404, USA (GS-IN-US-342-4494).
